# Is tDCS an Adjunct Ergogenic Resource for Improving Muscular Strength and Endurance Performance? A Systematic Review

**DOI:** 10.3389/fpsyg.2019.01127

**Published:** 2019-05-16

**Authors:** Sergio Machado, Petra Jansen, Victor Almeida, Jitka Veldema

**Affiliations:** ^1^Laboratory of Physical Activity Neuroscience, Physical Activity Sciences Postgraduate Program, Salgado de Oliveira University (UNIVERSO), Niterói, Brazil; ^2^Faculty of Psychology, Education and Sport Science, University of Regensburg, Regensburg, Germany; ^3^Helios Klinik Kipfenberg, Kipfenberg, Germany

**Keywords:** transcranial direct current stimulation, tDCS, muscular strength, endurance, systematic review

## Abstract

Exercise performance is influenced by many physical factors, such as muscle strength and endurance. Particularly in the physical fitness and sports performance contexts, there are many types of ergogenic aids to improve muscular strength and endurance performance, with non-athletes and even athletes using illegal drugs to reach the top. Thus, the development of innovative methods to aid in exercise performance is of great interest. One such method is transcranial direct current stimulation (tDCS). A systematic search was performed on the following databases, until January 2019; PubMed/MEDLINE, SCOPUS, and Pedro database. Studies on tDCS for muscular strength and endurance performance improvement in non-athletes and athletes adults were included. We compared the effect of anodal-tDCS (a-tDCS) to a sham/control condition on the outcomes muscular strength and endurance performance. We found 26 controlled trials. No trial mentions negative side effects of the intervention. The data show differences between the studies investigating muscle strength and the studies evaluating endurance, with regard to successful use of tDCS. Studies investigating the efficiency of tDCS on improving muscular strength demonstrate positive effects of a-tDCS in 66.7% of parameters tested. In contrast, in studies evaluating the effects of a-tDCS on improving endurance performance the a-tDCS revealed a significant improvement in only 50% of parameters assessed. The majority of the data shows consistently influence of a-tDCS on muscular strength, but not to endurance performance. The results of this systematic review suggest that a-tDCS can improve muscular strength, but not to endurance performance.

## Introduction

Exercise performance is influenced by many physical factors, such as muscle strength and endurance (Sleivert and Rowlands, 1996; Neumayr et al., 2003; McCormick et al., 2015). Particularly in the physical fitness and sports performance contexts, there are many types of ergogenic aids to improve muscular strength and endurance performance (Schubert and Astorino, 2012), with non-athletes and even athletes using illegal drugs to reach the top (Savulescu et al., 2004). Some years ago, sport scientists started to focus on the study of the brain as the central governor, and thus, regulates exercise with regards to a neurally calculated safe exertion by the body and how brain could limit or improve physical performance (Noakes, 2012). Since then, several studies investigated and showed the essential role of the brain in the determination of fatigue and muscular strength and endurance performance (Gandevia, 2001; Noakes, 2011a,b, 2012). Thus, the development of innovative methods to aid in exercise performance is of great interest (Noakes, 2012; Van Cutsem et al., 2017a). One such method is transcranial direct current stimulation (tDCS).

tDCS is a noninvasive technique that emits a weak electrical current that can promote excitation, through tonic depolarization of the membrane resting potential (anodic stimulus, a-tDCS), or cortical inhibition, by hyperpolarization of the membrane resting potential (cathodic stimulus, c-tDCS) (Nitsche and Paulus, 2000; Stagg and Nitsche, 2011), i.e., increase or decrease of spontaneous firing rate of neurons affected by the electrical current (Bikson et al., 2004; Rahman et al., 2013).

Last years, several researchers have begun to verify the effects of tDCS on physical performance in healthy individuals (Lattari et al., 2016, 2017, 2018b; Angius et al., 2018). tDCS can be used as a neuromodulatory ergogenic resource for healthy individuals to change physical performance, such as lead to an increase in muscular strength (Lattari et al., 2016, 2017, 2018b), and endurance (Okano et al., 2015; Lattari et al., 2018a), in both non-athletes (Okano et al., 2015; Lattari et al., 2016, 2017, 2018a,b; Angius et al., 2018) and athletes (Sales et al., 2016; Hazime et al., 2017; Vargas et al., 2018) that have been using tDCS during their training programs (Reardon, 2016; Edwards et al., 2017). In line with this, the objective of this systematic review was to verify whether tDCS is an effective ergogenic resource for muscular strength and endurance in non-athletes and athletes.

## Methods

The method of this study was designed and reported according to the recommendations of the Preferred Reporting Items for Systematic reviews and Meta-Analyses (PRISMA) (Green and Higgins, 2011) and the Cochrane Handbook for Systematic Reviews of Interventions (Liberati et al., 2009).

Studies were included according to Participants, Intervention, Comparison, Outcomes, and Setting (PICOS) inclusion criteria. Participants were healthy men and women adults, athletes, strength and endurance training practitioners or sedentary, with no history of mioarticular injury and no psychiatric illness. Intervention were used the effects of the anode stimulus of tDCS (a-tDCS). Comparators were sham tDCS (i.e., the placebo stimulus) or no interventions (i.e., control). Outcomes for both the muscular strength and endurance were evaluated from different points of view: (1) physical tasks that consist of uniarticular exercise or multiarticular exercise; (2) physical performance that was measured objectively as endurance time, total work performed, force production during a maximal voluntary contraction (MVC), peak power, mean power, and/or time to exhaustion. All these variables are considered the primary outcomes of our review. Study Design were only randomized and non-randomized trials, using either cross-over or parallel group designs, comparing an intervention encompassing a-tDCS with a sham group on muscle strength or no intervention.

We analyzed only studies published in English language. A systematic literature search was conducted between December 10−2018 and January 10−2019. The following databases were used: PubMed, ISI Web of Science, and Scopus. No filters were applied in the search.

The search was performed using the terms physical exercise, strength training, resistance training, endurance training, cycling, effort, physical exertion, fatigue, and athletic performance, individually combined with transcranial direct current stimulation and tDCS, in all databases.

Included important reports and reviews regarding tDCS and muscle strength or endurance were manually screened for additional relevant studies. Experts on the field, including authors from the reports, were also requested to suggest any additional trials in order to ensure that the review was as comprehensive and up-to-date as possible.

To facilitate the interpretation of our results, the findings were structured in two categories: (i) studies on the effects of tDCS on muscle strength performance (ii) studies on the effects of tDCS on endurance performance. This strategy was used due to the need to differ these physical tasks in terms of physiological responses (Sidhu et al., 2013). In addition, after revision of the studies, future perspectives for new researches were proposed based on the gaps in the existing literature and ethical and regulatory issues related to the use of tDCS as an enhancer for physical performance in athletes.

## Results

**Figure 1:**
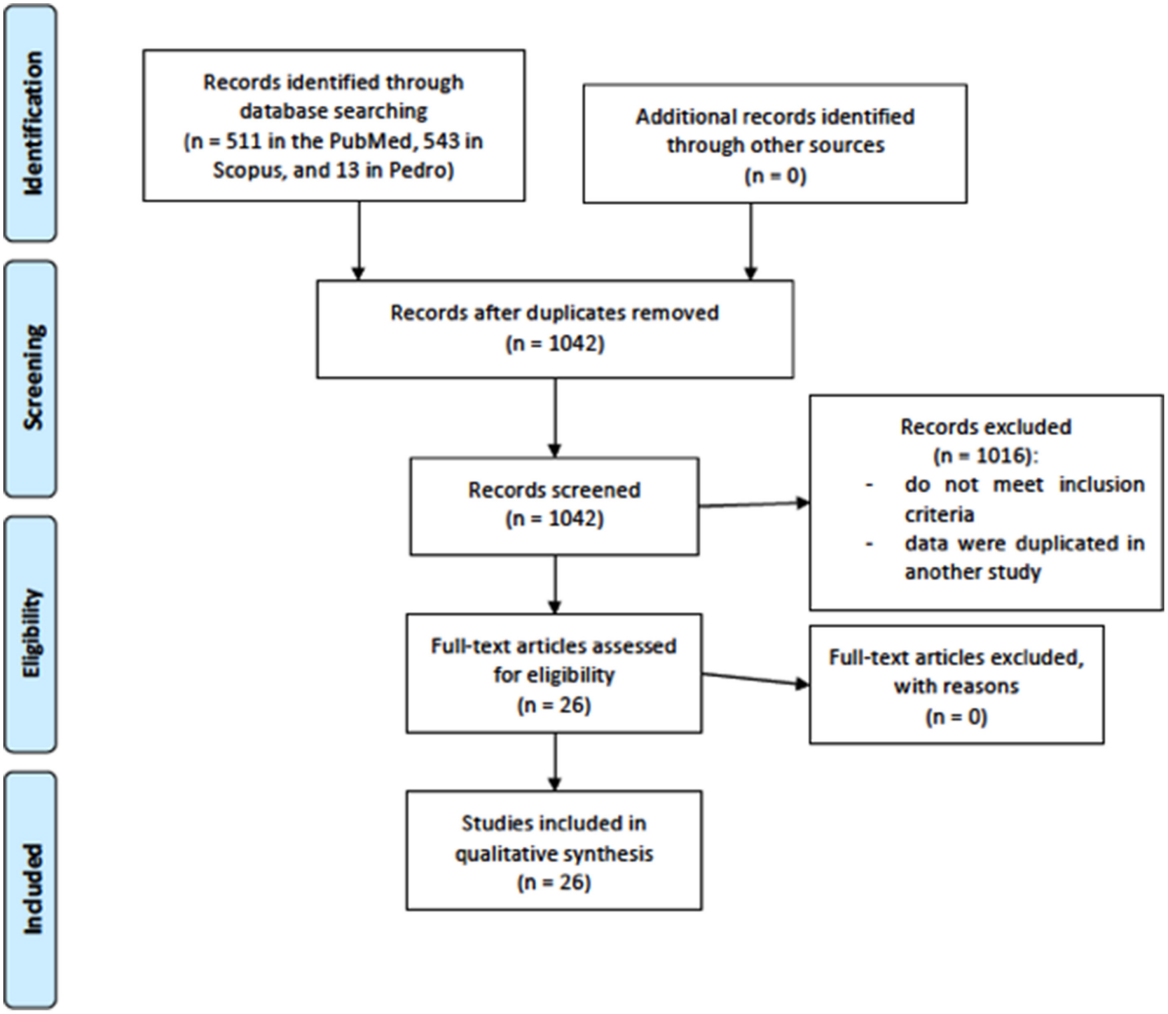
Flow chart for study selection.

The results identified a total of 1,067 articles (511 in the PubMed, 543 in Scopus, and 13 in Pedro). After the removal process of duplicate articles (*n* = 25), a total of 1,042 articles remained. One thousand sixteen articles were removed by title and/or abstract. After the removal process, 26 articles were included for systematic review, 18 examining the effects of tDCS on muscular strength performance and 8 on endurance performance. Flow chart is presented in Figure 1.

### tDCS for Improving Strength Performance

A total of 18 studies (Cogiamanian et al., 2007; Tanaka et al., 2009; Kan et al., 2013; Williams et al., 2013; Hendy and Kidgell, 2014; Montenegro et al., 2015; Abdelmoula et al., 2016; Angius et al., 2016; Lattari et al., 2016, 2017, 2018b; Sales et al., 2016; Flood et al., 2017; Frazer et al., 2017; Hazime et al., 2017; Radel et al., 2017; Ciccone et al., 2018; Vargas et al., 2018) evaluated the efficiency of tDCS for improving muscular strength performance (see Tables 1, 2). No relevant side effects occurred.

**Table 1:** Study characteristics of muscular strength exercises.

**References**	***N***	**Drop-outs (N;%)**	**Gender**	**Age**	**Experience with ST**	**Mode of tDCS application**	**Design**
Lattari et al., 2016	a-tDCS = 10 sham = 10	None	a-tDCS = 10 (M) sham = 10 (M)	26.5 (±5.0)	>6 months	Offline	Crossover
Lattari et al., 2017	a-tDCS = 10 sham = 10	None	a-tDCS = 10 (M) sham = 10 (M)	22.1 (±3.8)	47.8 ± 22.7 months	Offline	Crossover
Lattari et al., 2018b	a-tDCS = 15 sham = 15	None	a-tDCS = 15 (F) sham = 15 (F)	24.5 (±3.3)	>1 year	Offline	Crossover
Hazime et al., 2017	a-tDCS = 8 sham = 8	None	a-tDCS = 8 (F) sham = 8 (F)	19.7 (±2.3)	Handball athletes (31 weeks of ST)	Offline	Crossover
Vargas et al., 2018	a-tDCS = 20 sham = 20	None	a-tDCS = 20 (F) sham = 20 (F)	16.1 (±0.9)	>5 years of training in soccer (not reported with ST)	Online	Crossover
Sales et al., 2016	a-tDCS = 19 sham = 19	None	a-tDCS = 19 (M) sham = 19 (M)	25.1 (±3.9)	Physically active (not reported with ST)	Online	Crossover
Cogiamanian et al., 2007	a-tDCS = 9 control = 15	None	a-tDCS = 5 (F) and 4 (M) control = 9 (F) and 6 (M)	24.3	None of the subjects were engaged in competitive sport activities specifically involving elbow flexor muscles	Offline	Parallel
Frazer et al., 2017	a-tDCS = 13 sham = 13	None	a-tDCS = 5 (F) and 8 (M) sham = 5 (F) and 8 (M)	18–35	Not reported	Offline	Crossover
Hendy and Kidgell, 2014	a-tDCS = 10 sham = 10	None	a-tDCS = 5 (F) and 5 (M) sham = 5 (F) and 5 (M)	25.9 ± 1.3	Not reported	Online	Crossover
Williams et al., 2013	a-tDCS = 18 sham = 18	None	a-tDCS = 9 (M) and 9 (F) sham = 9 (M) and 9 (F)	25 ± 6	9 active/9 low active	Offline	Crossover
Angius et al., 2016	a-tDCS = 9 sham = 9	None	a-tDCS = 9 (M) sham = 9 (M)	23.0 (±2.0)	Recreationally active (not reported with ST)	Offline	Crossover
Kan et al., 2013	a-tDCS = 15 sham = 15	None	a-tDCS = 15 (M) sham = 15 (M)	27.7 (±8.4)	Not reported	Offline	Crossover
Abdelmoula et al., 2016	a-tDCS = 11 sham = 11	None	a-tDCS = 3 (F) and 8 (M) sham = 3 (F) and 8 (M)	25.0 ± 1.8	Not reported	Offline	Crossover
Tanaka et al., 2009	a-tDCS = 10 sham = 10	2 (20%)	a-tDCS = 8 (M) and 2 (F) sham = 8 (M) and 2 (F)	23.8 (20–35)	Not reported	Online	Crossover
Montenegro et al., 2015	a-tDCS = 14 sham = 14	None	a-tDCS = 14 (M) sham = 14 (M)	26.0 (±4.0)	> 6 months	Offline	Crossover
Flood et al., 2017	a-tDCS = 12 sham = 12	None	a-tDCS = 12 (M) sham = 12 (M)	24.4 ± 3.8	Recreationally active	Offline	Crossover
Ciccone et al., 2018	a-tDCS = 20 sham = 20	None	a-tDCS = 10 (M) and 10 (F) sham = 10 (M) and 10 (F)	21.0 (±1.5)	Recreationally active (not reported with ST)	Online	Crossover
Radel et al., 2017	a-tDCS = 22 sham = 22	None	a-tDCS = 13 (M) and 9 (F) sham = 13 (M) and 9 (F)	21.3 ± 0.4	Not reported	Online	Crossover

*N, number of participants; M, male; F, female; %, percentage; ST, Strength training; >, greater*.

**Table 2:** Study protocols for muscular strength exercises.

**References**	**Intervention protocol (a-tDCS)**				**Control**	**Strength exercise characteristic**			**Main outcomes**
	**Montage of electrodes**	**Electrode size (cm^**2**^)**	**Current intensity (mA)**	**Duration (min)**		**Muscle investigated**	**Type of contraction**	**Muscle strength test**	
Lattari et al., 2016	Left DLPFC (stimulus) and right OFC (reference)	35 (stimulus and reference)	2	20	30 (s) (sham)	Elbow flexors	Dynamic	Volume-load (kg)	a-tDCS > sham (*p* < 0.05)
Lattari et al., 2017	Central MC (stimulus) and right OFC (reference)	35 (stimulus and reference)	2	20	30 (s) (sham)	Ankle, hip, and knee extensors	Dynamic	Muscle power (W)	≠ between the conditions
Lattari et al., 2018b	Left DLPFC (stimulus) and right OFC (reference)	35 (stimulus and reference)	2	20	30 (s) (sham)	Ankle, hip, and knee extensors	Dynamic	Volume-load (kg)	a-tDCS > sham (*p* < 0.05)
Hazime et al., 2017	MC dominant limb (stimulus) and ipsilateral OFC (reference)	35 (stimulus and reference)	2	20	30 (s) (sham)	Internal and external rotator	Isometric	MIVC (N/kg)	MIVC (internal and external rotator): a-tDCS > sham (*p* < 0.05)
Vargas et al., 2018	Left and right MC (stimulus) and ipsilateral OFC (reference)	35 (stimulus and reference)	2	20	30 (s) (sham)	Knee extensors	Isometric	MIVC (N/kg) in dominant and non-dominant limb	Dominant a-tDCS > sham (*p* < 0.05) Non-dominant ≠ between the conditions
Sales et al., 2016	Left TC (stimulus) and right OFC (reference)	35 (stimulus and reference)	2	20	30 (s) (sham)	Knee extensors	Dynamic	Isokinetic testing (angular velocity of 180°·s^−1^ and 60°·s^−1^):Total work (J) and peak torque (N.m)	Total work: a-tDCS > sham (*p* < 0.05) Peak torque: ≠ between the conditions
Cogiamanian et al., 2007	Right MC (stimulus) and right shoulder (reference)	35 (stimulus and reference)	1.5	10	No intervention	Left elbow flexors	Isometric	MIVC (N) and TTE with 35% of the MIVC (s)	MIVC: ≠ between the conditions TTE: a-tDCS > control (p < 0.05)
Frazer et al., 2017	Left MC (stimulus) and right OFC (reference)	25	2	20	30 (s) (sham)	Left biceps brachii	Dynamic	1RM	↑ Strength of the untrained limb
Hendy and Kidgell, 2014	Left MC (stimulus) and Left OFC (reference)	25	2	20	30 (s) (sham)	Wrist extensor muscles	Dynamic	1RM	↑ Strength of untrained wrist extensor muscles
Williams et al., 2013	Right MC (stimulus) and left OFC	35	1.5	20	30 (s) (sham)	Left elbow flexors	Isometric	MVC and TTF at 20% MVC	TTE: ↑ Endurance time
Angius et al., 2016	Two montages of the electrodes: First: Left MC (stimulus) and right OFC (reference); Second: Left MC (stimulus) and left shoulder (reference)	12 (stimulus and reference)	2	10	30 (s) (sham)	Right knee extensors	Isometric	MIVC (N.m) TTE with 20% of the MIVC (s)	MIVC: NR TTE: Second position-a-tDCS > sham (p < 0.05); First position ≠ between the conditions
Kan et al., 2013	Right MC (stimulus) and right shoulder (reference)	24 (stimulus and reference)	2	10	30 (s) (sham)	Left elbow flexors	Isometric	MIVC (N.m) and TTE with 30% of the MIVC (s)	MIVC: ≠ between the conditions TTE: ≠ between the conditions
Abdelmoula et al., 2016	Left MC (stimulus) and right shoulder (reference)	35	1.5	10	90 (s) (sham)	Left elbow flexors	Isometric	MIVC (N) and TTE at 35% MVC	TTE: ↑ Endurance time
Tanaka et al., 2009	Right MC (stimulus) and right OFC (reference)	35 (stimulus and reference)	2	10	30 (s) (sham)	Adduction between the left great toe and the digitus secundus (leg pinch force) adduction Between the Index finger and the Thumb pad of the Left hand (hand pinch force)	Isometric	PF (N)	PF (Leg): a-tDCS > sham (*p* < 0.01)
Montenegro et al., 2015	Left MC (stimulus) and right OFC (reference)	35 (stimulus and reference)	2	20	30 (s) (sham)	Knee extensors and flexors	Dynamic	Isokinetic testing (angularvelocity of 60°·s^−1^:Total work (J) and peak torque (N.m)	Total work: ≠ between the conditions Peak torque: ≠ between the conditions
Flood et al., 2017	Positioning of the electrodes (4 × 1) MC contralateral to the non-dominant side (stimulus, C3 or C4) and four cathodal electrodes placed at a distance of 5 cm around the anode (reference); radius ≈ 1.1 cm	−	2	20	At the start and at the end (2 mA in ramping)	Elbow flexors	Isometric	MIVC (N.m) and TTE at 30% MVC	No improvement
Ciccone et al., 2018	Two montages of electrodes: First- Left TC (stimulus) and right OFC (reference); Second- Right TC (stimulus) and left OFC (reference)	25 (stimulus and reference)	2	20	30 (s) (sham)	Knee extensors	Dynamic	Isokinetic testing (angular velocity of 180°·s^−1^): Average work (Nm.s)	≠ between the conditions
Radel et al., 2017	Two montages of electrodes (4x1): First: Right MC (stimulus) and four cathodal electrodes placed at a distance of 4 cm around the anode (reference); Second: Right DLPFC (stimulus) and four cathodal electrodes placed at a distance of 4 cm around the anode (reference); radius ≈1.1 cm	-	2	≤ 20	30 (s) (sham)	Elbow flexors	Isometric	TTE at 35% MVC	No improvement

*↑ (increased); maximal dynamic strength (1RM); maximal voluntary contraction (MVC); peak power output (PPO); a-tDCS- anodal transcranial direct current stimulation; CG, control group; cm^2^, square centimeter; mA, milliamps; min, minutes; s, seconds; MC, motor cortex; DLPFC, dorsolateral prefrontal cortex; OBF, orbitofrontal cortex; ≈, approximately; MIVC, Maximal Isometric Voluntary Contraction; PF, Pinch Force; N, Newtons; N.m, Newtons per meter; N/kg, Newtons per kilogram (normalized by the body mass of each participant); Kg, Kilogram; J, Joules; %, percentage; Nm.s, Newtons meter per second; TTF, Time to failure; TTE, time to exhaustion; NR, not reported*.

### Study Characteristics

In total, 496 participants, 282 males, and 214 females were included in the 19 studies. The mean age of the participants from studies varied between 16.01 (±0.9) (Vargas et al., 2018) and 27.7 (±8.4) (Kan et al., 2013) years. Regarding tDCS conditions, a-tDCS condition had sample sizes between 8 (Hazime et al., 2017) and 22 (Radel et al., 2017), with a total of 245 participants among studies, while control condition had sample sizes between 8 (Hazime et al., 2017) and 22 (Radel et al., 2017), with a total of 251 individuals among studies. Two studies had dropouts, 20% (2 participants) in the Tanaka's study (Tanaka et al., 2009) and 4.5% (1 participant) in the Radel's study (Radel et al., 2017). As expected, most studies had more male than female participants. In addition, only five studies (Montenegro et al., 2015; Lattari et al., 2016, 2017, 2018b; Hazime et al., 2017) reported experience in ST, which could be an influencer factor in a-tDCS response. Regarding mode of tDCS application, twelve studies using offline mode (Cogiamanian et al., 2007; Kan et al., 2013; Williams et al., 2013; Montenegro et al., 2015; Abdelmoula et al., 2016; Angius et al., 2016; Lattari et al., 2016, 2017, 2018b; Flood et al., 2017; Frazer et al., 2017; Hazime et al., 2017), while six using online mode (Tanaka et al., 2009; Hendy and Kidgell, 2014; Sales et al., 2016; Radel et al., 2017; Ciccone et al., 2018; Vargas et al., 2018). Almost studies used crossover study design (Tanaka et al., 2009; Kan et al., 2013; Williams et al., 2013; Hendy and Kidgell, 2014; Montenegro et al., 2015; Abdelmoula et al., 2016; Angius et al., 2016; Lattari et al., 2016, 2017, 2018b; Sales et al., 2016; Flood et al., 2017; Frazer et al., 2017; Hazime et al., 2017; Radel et al., 2017; Ciccone et al., 2018; Vargas et al., 2018), and only one study integrated a parallel group study design (Cogiamanian et al., 2007), with only a single session of tDCS for both of them.

### Study Protocols for Muscular Strength Exercises

All studies tested anodal tDCS in comparison to sham tDCS. Concerning tDCS conditions, a-tDCS protocol delivered stimulation on motor cortex (MC) (Cogiamanian et al., 2007; Tanaka et al., 2009; Kan et al., 2013; Williams et al., 2013; Hendy and Kidgell, 2014; Montenegro et al., 2015; Abdelmoula et al., 2016; Angius et al., 2016; Flood et al., 2017; Frazer et al., 2017; Hazime et al., 2017; Lattari et al., 2017; Radel et al., 2017; Ciccone et al., 2018; Vargas et al., 2018), dorsolateral prefrontal cortex (DLPFC) (Lattari et al., 2016, 2018b; Radel et al., 2017), and temporal cortex (TC) (Sales et al., 2016; Ciccone et al., 2018). Two studies used high-definition tDCS (Flood et al., 2017; Radel et al., 2017). The montage of electrodes respected a 4 × 1 ring configuration with the central electrode located over the hand cortical area (anodal) and return electrodes placed in a ring around the central anode (cathodal) at a radius around 5 and 4 cm (Flood et al., 2017; Radel et al., 2017). Electrodes with different sizes, i.e., between 12 and 35 cm^2^, were used in the target areas (Cogiamanian et al., 2007; Tanaka et al., 2009; Kan et al., 2013; Williams et al., 2013; Hendy and Kidgell, 2014; Montenegro et al., 2015; Abdelmoula et al., 2016; Angius et al., 2016; Lattari et al., 2016, 2017, 2018b; Sales et al., 2016; Frazer et al., 2017; Hazime et al., 2017; Ciccone et al., 2018; Vargas et al., 2018). In relation to the electrodes of 4 X 1 ring configuration, the estimated diameter was 1.1 cm (Flood et al., 2017; Radel et al., 2017). Three studies applied an intensity of 1.5 mA (Cogiamanian et al., 2007; Williams et al., 2013; Abdelmoula et al., 2016) and the others used an intensity of 2 mA (Tanaka et al., 2009; Kan et al., 2013; Hendy and Kidgell, 2014; Montenegro et al., 2015; Angius et al., 2016; Lattari et al., 2016, 2017, 2018b; Sales et al., 2016; Flood et al., 2017; Frazer et al., 2017; Hazime et al., 2017; Radel et al., 2017; Ciccone et al., 2018; Vargas et al., 2018). In addition, session duration varied between 10 (Cogiamanian et al., 2007; Tanaka et al., 2009; Kan et al., 2013; Abdelmoula et al., 2016; Angius et al., 2016) and 20 min (Williams et al., 2013; Hendy and Kidgell, 2014; Montenegro et al., 2015; Lattari et al., 2016, 2017, 2018b; Sales et al., 2016; Flood et al., 2017; Frazer et al., 2017; Hazime et al., 2017; Radel et al., 2017; Ciccone et al., 2018; Vargas et al., 2018). With regard to control conditions, just one study applied no placebo stimulus (sham) (Cogiamanian et al., 2007) and the others used the sham condition (Tanaka et al., 2009; Kan et al., 2013; Williams et al., 2013; Hendy and Kidgell, 2014; Montenegro et al., 2015; Abdelmoula et al., 2016; Angius et al., 2016; Lattari et al., 2016, 2017, 2018b; Sales et al., 2016; Flood et al., 2017; Frazer et al., 2017; Hazime et al., 2017; Radel et al., 2017; Ciccone et al., 2018; Vargas et al., 2018). Fifteen studies utilized a 30 s period as sham stimulus (Tanaka et al., 2009; Kan et al., 2013; Williams et al., 2013; Hendy and Kidgell, 2014; Montenegro et al., 2015; Angius et al., 2016; Lattari et al., 2016, 2017, 2018b; Sales et al., 2016; Frazer et al., 2017; Hazime et al., 2017; Radel et al., 2017; Ciccone et al., 2018; Vargas et al., 2018) and three used other types of sham/control condition (Cogiamanian et al., 2007; Abdelmoula et al., 2016; Flood et al., 2017). The montage of the electrodes was the same as the a-tDCS condition.

The muscular strength exercise characteristics demonstrated that isometric (Cogiamanian et al., 2007; Tanaka et al., 2009; Kan et al., 2013; Abdelmoula et al., 2016; Angius et al., 2016; Flood et al., 2017; Hazime et al., 2017; Radel et al., 2017; Vargas et al., 2018) and dynamic contractions (Hendy and Kidgell, 2014; Montenegro et al., 2015; Lattari et al., 2016, 2017, 2018b; Sales et al., 2016; Frazer et al., 2017; Ciccone et al., 2018) were investigated. In dynamic exercises several types of tests were used, such as isokinetic testing (Montenegro et al., 2015; Sales et al., 2016; Ciccone et al., 2018), contractions against constant load (Hendy and Kidgell, 2014; Lattari et al., 2016, 2018b; Frazer et al., 2017), and muscular power (Lattari et al., 2017). Studies investigated the following muscles: elbow flexors (Cogiamanian et al., 2007; Kan et al., 2013; Williams et al., 2013; Abdelmoula et al., 2016; Lattari et al., 2016; Flood et al., 2017; Radel et al., 2017), internal and external rotator (Hazime et al., 2017), knee extensors (Montenegro et al., 2015; Angius et al., 2016; Sales et al., 2016; Lattari et al., 2017, 2018b; Ciccone et al., 2018; Vargas et al., 2018), adduction between the left great toe and the digitus secundus and adduction between the index finger and the thumb pad of the left hand (Tanaka et al., 2009), ankle, hip, and knee extensors (Lattari et al., 2017, 2018b), knee extensors and flexors (Montenegro et al., 2015). Alterations in muscular strength were examined through muscular endurance (Cogiamanian et al., 2007; Kan et al., 2013; Williams et al., 2013; Montenegro et al., 2015; Abdelmoula et al., 2016; Angius et al., 2016; Lattari et al., 2016, 2018b; Sales et al., 2016; Flood et al., 2017; Radel et al., 2017; Ciccone et al., 2018), and maximal strength tests (Cogiamanian et al., 2007; Tanaka et al., 2009; Kan et al., 2013; Williams et al., 2013; Hendy and Kidgell, 2014; Abdelmoula et al., 2016; Angius et al., 2016; Flood et al., 2017; Frazer et al., 2017; Hazime et al., 2017; Vargas et al., 2018). Just one study examined the effects of a single session of tDCS on the muscular power (Lattari et al., 2017).

### The Effectiveness of tDCS on Improving the Muscular Strength Performance

Regarding maximal voluntry contraction (MVC), two studies showed difference between a-tDCS and sham conditions (Hendy and Kidgell, 2014; Frazer et al., 2017). Both studies observed an increase in strength of untrained limbs. When analyzed the maximal isometric voluntary contractions (MIVC), no difference was observed between a-tDCS and sham conditions in 4 studies (Cogiamanian et al., 2007; Kan et al., 2013; Abdelmoula et al., 2016; Flood et al., 2017). Three studies showed a-tDCS was superior to sham condition in increase MIVC (Tanaka et al., 2009; Hazime et al., 2017; Vargas et al., 2018). The first in shoulder internal and external rotators of the shoulder (Hazime et al., 2017), the second in knee extensors (Vargas et al., 2018) and the third in the adduction between the left great toe and the digitus secundus (Tanaka et al., 2009). Regarding muscular endurance, there were finding significant differences between a-tDCS and sham conditions in seven studies (Cogiamanian et al., 2007; Williams et al., 2013; Abdelmoula et al., 2016; Angius et al., 2016; Lattari et al., 2016, 2018b; Sales et al., 2016). These differences were observed in isometric contraction (Cogiamanian et al., 2007; Williams et al., 2013; Abdelmoula et al., 2016; Angius et al., 2016), muscle action against a constant load (Lattari et al., 2016, 2018b) and isokinetic (Sales et al., 2016) strength tests. Six studies do not reveal significant differences between a-tDCS and sham conditions for muscular endurance in isometric contraction (Kan et al., 2013; Angius et al., 2016; Flood et al., 2017; Radel et al., 2017), and isokinetic (Montenegro et al., 2015; Ciccone et al., 2018) strength tests. For muscle power, one study was performed and showed that there was no significant difference between a-tDCS and sham condition (Lattari et al., 2017).

### tDCS for Improving Endurance Performance

Eight studies (Angius et al., 2015, 2018; Okano et al., 2015; Vitor-Costa et al., 2015; Barwood et al., 2016; Sasada et al., 2017; Lattari et al., 2018a; Holgado et al., 2019) evaluated the efficiency of tDCS for improving the endurance (see Tables 3, 4). Relevant side effects were not described.

**Table 3:** Study characteristics of endurance exercises.

**References**	***N***	**Drop-outs (N;%)**	**Gender**	**Age**	**Experience with ET**	**Mode of tDCS application**	**Design**
Angius et al., 2018	a-tDCS = 12 sham = 12	None	a-tDCS = 4 (F) and 8 (M) sham = 4 (F) and 8 (M)	24 ± 5	Recreationally active	Offline	Crossover
Okano et al., 2015	a-tDCS = 10 sham = 10	None	a-tDCS = 10 (M) sham = 10 (M)	33 ± 9	Athletes (cyclists)	Offline	Crossover
Lattari et al., 2018a	a-tDCS = 11 sham = 11	None	a-tDCS = 11 (F) sham = 11 (F)	24 ± 2.2	Moderately active	Offline	Crossover
Vitor-Costa et al., 2015	a-tDCS = 11 sham = 11	None	a-tDCS = 11 (M) sham = 11 (M)	26 ± 4	Physically active	Offline	Crossover
Angius et al., 2015	Study 1: a-tDCS = 9 sham = 9 control = 9 Study 2: a-tDCS = 7 sham = 7 control = 7	None	Study 1: a-tDCS = 9 (M) sham = 9 (M) control = 9 (M) Study 2: a-tDCS = 7 (M) sham = 7 (M) control = 7 (M)	23 ± 4	Recreationally active	Offline	Crossover
Barwood et al., 2016	Study 1: a-tDCS = 6 sham = 6 Study 2: a-tDCS = 8 sham = 8	None	Study 1: a-tDCS = 6 (M) sham = 6 (M) Study 2: a-tDCS = 8 (M) sham = 8 (M)	Study 1: 21 ± 2 Study 2: 21 ± 1	Physically active	Offline	Crossover
Sasada et al., 2017	a-tDCS = 23 sham = 23	None	a-tDCS = 6 (F) and 17 (M) sham = 6 (F) and 17 (M)	21–30	Athletes (various)	Offline	Crossover
Holgado et al., 2019	a-tDCS = 36 sham = 36	None	a-tDCS = 36 (M) sham = 36 (M)	27 ± 6.8	Not reported	Offline	Crossover

*N, number of participants; M, male; F, female; %, percentage; ET, Endurance training; >, greater*.

**Table 4:** Study protocols from endurance exercises.

**References**	**Intervention protocol (a-tDCS)**				**Control**	**Endurance exercise characteristic**		**Main outcomes**
	**Montage of electrodes**	**Electrode size (cm^**2**^)**	**Current intensity (mA)**	**Duration (min)**		**Muscle investigated**	**Exercise protocol**	
Angius et al., 2018	Central MC (stimulus) and both ipsilateral shoulder (reference)	35 (stimulus and reference)	2	10	30 (s) Sham	Lower limbs	Cycling TTF at 70%PPO	↑ Endurance time
Okano et al., 2015	Left TC (stimulus) and Right OBF (reference)	35 (stimulus and reference)	2	20	30 (s) Sham	Lower limbs	Maximal incremental cycling test. From 15W+25W min^_1^	↑ PPO
Lattari et al., 2018a	Left DLPFC (stimulus) and Right OBF (reference)	35 (stimulus and reference)	2	20	30 (s) Sham	Lower limbs	Cycling TTE at 100% PPO	↑ Endurance time
Vitor-Costa et al., 2015	Central MC (stimulus) and occipital protuberance (reference)	36 (stimulus) and 35 (reference)	2	13	30 (s) Sham	Lower limbs	Cycling TTE at 80% PPO	↑ Endurance time
Angius et al., 2015	Left MC (stimulus) and Right DLPFC (reference)	12 (stimulus and reference)	2	10	30 (s) Sham and Control	Lower limbs	Cycling TTE at 70% PPO	No improvement
Barwood et al., 2016	Left TC (stimulus) and Right OBF (reference)	35 (stimulus and reference)	2	20	30 (s) Sham	Lower limbs	Study 1: 20 km TT; Study 2: TTE at 75% PPO	No improvement
Sasada et al., 2017	Central MC (stimulus) and Right OBF (reference)	NS	2	15	30 (s) Sham	Lower limbs	Cycling, 30 s maximal-effort sprint cycling	No improvements
Holgado et al., 2019	Left DLPFC (stimulus) and Contralateral shoulder (reference)	25 (stimulus and reference)	2	20	30 (s) Sham	Lower limbs	Cycling, 20-min TT	No improvements

*↑ increased; time to exhaustion (TTE); time to failure (TTF); time trial (TT); Power output (PO); peak power output (PPO); a-tDCS, anodal transcranial direct current stimulation; cm^2^, square centimeter; mA-milliamps; min, minutes; s, seconds; MC, motor cortex; DLPFC, dorsolateral prefrontal cortex; OBF, orbitofrontal cortex; W, watts; %, percentage; NR, not reported*.

### Study Characteristics

In total, 280 participants, 240 males and 40 females were included in the 8 studies. The mean age of the participants from studies varied between 21 (Sasada et al., 2017) and 33(±9) (Okano et al., 2015) years. Regarding tDCS conditions, a-tDCS condition had sample sizes between 6 (Barwood et al., 2016) and 36 (Holgado et al., 2019), with a total of 133 participants among studies, while control condition had sample sizes between 6 (Barwood et al., 2016) and 36 (Holgado et al., 2019), with a total of 147 individuals among studies. There was no dropout. As expected, most studies had more female than male participants. In addition, only two studies (Okano et al., 2015; Sasada et al., 2017) reported experience in ET, which could be an influencer factor in a-tDCS response. Regarding mode of tDCS application, all studies using offline mode (Angius et al., 2015, 2018; Okano et al., 2015; Vitor-Costa et al., 2015; Barwood et al., 2016; Sasada et al., 2017; Lattari et al., 2018a; Holgado et al., 2019). In addition, all studies used crossover study design (Angius et al., 2015, 2018; Okano et al., 2015; Vitor-Costa et al., 2015; Barwood et al., 2016; Sasada et al., 2017; Lattari et al., 2018a; Holgado et al., 2019), with only a single session of tDCS for all of them.

### Study Protocols for Endurance Exercises

All studies tested anodal tDCS in comparison to sham tDCS. Concerning tDCS conditions, a-tDCS protocol delivered stimulation on motor cortex (MC) (Angius et al., 2015, 2018; Vitor-Costa et al., 2015; Sasada et al., 2017), dorsolateral prefrontal cortex (DLPFC) (Lattari et al., 2018a; Holgado et al., 2019), and temporal cortex (TC) (Okano et al., 2015; Barwood et al., 2016). Electrodes with different sizes, i.e., between 12 and 36 cm^2^, were used in the target areas. All studies applied an intensity of 2 mA (Angius et al., 2015, 2018; Okano et al., 2015; Vitor-Costa et al., 2015; Barwood et al., 2016; Sasada et al., 2017; Lattari et al., 2018a; Holgado et al., 2019). In addition, two studies had session duration of 10 (Angius et al., 2015, 2018), one of 13 (Vitor-Costa et al., 2015), one of 15 (Sasada et al., 2017), and four of 20 min (Okano et al., 2015; Barwood et al., 2016; Lattari et al., 2018a; Holgado et al., 2019). With regard to control conditions, just one study applied no placebo stimulus plus sham (Angius et al., 2015) and the others used just the sham condition (Okano et al., 2015; Vitor-Costa et al., 2015; Barwood et al., 2016; Sasada et al., 2017; Angius et al., 2018; Lattari et al., 2018a; Holgado et al., 2019). All studies utilized a 30 s period as sham stimulus (Angius et al., 2015, 2018; Okano et al., 2015; Vitor-Costa et al., 2015; Barwood et al., 2016; Sasada et al., 2017; Lattari et al., 2018a; Holgado et al., 2019). The montage of the electrodes was the same as the a-tDCS condition.

The endurance exercise characteristics demonstrated that only cycling exercises (Angius et al., 2015, 2018; Okano et al., 2015; Vitor-Costa et al., 2015; Barwood et al., 2016; Sasada et al., 2017; Lattari et al., 2018a; Holgado et al., 2019) were used, and studied just lower limbs (Angius et al., 2015, 2018; Okano et al., 2015; Vitor-Costa et al., 2015; Barwood et al., 2016; Sasada et al., 2017; Lattari et al., 2018a; Holgado et al., 2019). The changes in endurance were investigated through maximal incremental test (Okano et al., 2015), 30 s maximal-effort sprint cycling test (Sasada et al., 2017), time-trial (TT) (Barwood et al., 2016; Holgado et al., 2019), time to exhaustion (TTE) (Angius et al., 2015; Vitor-Costa et al., 2015; Barwood et al., 2016; Lattari et al., 2018a), time to fatigue (TTF) (Angius et al., 2018) assessments.

### The Effectiveness of tDCS on Improving the Endurance Performance

Regarding maximal incremental test, just one study investigated the effects of a-tDCS on endurance performance (Okano et al., 2015), and showed significant difference between a-tDCS and sham condition, with an increase in peak power output (PPO) after a-tDCS. Another study examined the effects of a-tDCS on 30 s maximal-effort sprint cycling test, revealed no significant difference between a-tDCS and sham condition (Sasada et al., 2017). Four articles examined the effects of a-tDCS on TTE (Angius et al., 2015; Vitor-Costa et al., 2015; Barwood et al., 2016; Lattari et al., 2018a). Two studies showed significant differences between a-tDCS and sham (Vitor-Costa et al., 2015; Lattari et al., 2018a), with increase in endurance time after a-tDCS, while two study did not reveal significant difference (Angius et al., 2015; Barwood et al., 2016). Two studies investigated the effects of a-tDCS on TT (Barwood et al., 2016; Holgado et al., 2019), with no study demonstrating significant difference between a-tDCS and sham condition. In addition, one study evaluated the effects of a-tDCS on TTF, with significant difference between a-tDCS and sham conditions. The authors showed an increase in endurance time after a-tDCS (Angius et al., 2018).

## Discussion

This review aimed to discuss the potential effects of tDCS as an ergogenic resource for muscular strength and endurance performance. The data of 26 controlled trials were analyzed (see Tables 1, 2). No trial mentions negative side effects of the intervention. The data show differences between the studies investigating muscle strength and the studies evaluating endurance, with regard to successful use of tDCS. Studies investigating the efficiency of tDCS on improving muscular strength demonstrate positive effects of a-tDCS in 66.7% of parameters tested. In contrast, in studies evaluating the effects of a-tDCS on improving endurance performance the a-tDCS revealed a significant improvement in only 50% of parameters assessed. The majority of the data shows consistently no influence of a-tDCS on muscular strength, but not to endurance performance. We will also discuss the potential directions of futures studies.

Due to the complex process which is the exercise practice, several brain areas may be involved in exercise regulation/limitation, and thus, a justification for the use of tDCS for performance improvement. However, most studies on tDCS and exercise performance and sports are not clear with respect to their hypotheses of why applying tDCS in a particular area of the brain for improving performance, such as the primary motor cortex (M1), the dorsolateral prefrontal cortex (CPFDL), and the insular cortex (IC).

Regarding brain areas, M1 is the most related to exercise performance due to its role in motor execution. Studies have consistently shown that central fatigue can compromise the physical performance of exercises of small muscle groups (e.g., elbow flexion), as well as exercises of large muscle groups (e.g., cycling). Specifically, spinal and supraspinal factors, such as reduced excitability of the motorneuron pool and the inability or limited ability of M1 and other supraspinal areas to increase the neural drive to compensate for this decrease in spinal excitability leads to decreased muscle capacity to produce strength/power and thus cause fatigue (Gandevia, 2001; Taylor and Gandevia, 2008; Taylor et al., 2016). Therefore, a reason to use tDCS over M1 would increase the excitability of it, which could result in sustained neural activity to the motor neuron, delay in the decrease of the neural unit to the active muscle and thus improve performance. In addition, other possible reasons for the application of tDCS over M1 could be modulate the pain perception. However, this mechanism still is unclear. A possible reason to direct M1 to pain modulation would be due to its connections with the insula and thalamus, as demonstrated in animal studies (Stepniewska et al., 1994). In addition, the a-tDCS in M1 increases the sensory and pain thresholds in healthy individuals as well as the level of pain in chronic pain patients (Vaseghi et al., 2014). In this regarding, it is suggested that exercise-induced pain plays a fundamental role in the regulation of performance, where individuals with better ability to tolerate or overcome pain would be more successful (Mauger, 2013). Therefore, the application of tDCS in M1 can also improve performance through exercise-induced pain attenuation.

With regard to PFC, whose main function is the cognitive control of behavior, seems to play an important role in processing internal and external cues related to the exercise performed (Robertson and Marino, 2016). PFC exerts a top-down influence that can result in changes of rhythm to complete the task, with prolongation of the motor output, slowing up the end of the exercise or the shutdown of the motor units, causing the end of the exercise (Robertson and Marino, 2016). Thus, the psychobiological model proposes this task of disengagement (that is, the end of the exercise) as a decision-making process based on the effort that depends on the motivation (for example, the maximal effort that a person is willing to exercise), perception of effort, knowledge of the endpoint of the exercise and distance/time remaining, and previous experience/memory of effort perception during exercise varying intensity and duration (Pageaux, 2014). A systematic review has confirmed that interventions aimed at decreasing the ability of PFC to exert control over bodily signals during exercise, such as mental fatigue (e.g., performing a cognitively prolonged task) may reduce endurance performance (Van Cutsem et al., 2017b). In fact, what has been observed is that there is a decrease in PFC oxygenation before the initiation of fatigue (Rupp and Perrey, 2008; Rooks et al., 2010). Therefore, the application of tDCS in the PFC could strengthen the ability of this region to disregard interoceptive cues (i.e., body signals), keeping the volitional drive to M1 and thus delaying the disengagement of the task (i.e., at the end of the exercise).

Another target area of tDCS studies on physical performance is the insular cortex (IC), considered as a responsible for cardiac autonomic control. Several types of studies indicate that the right IC is responsible for sympathetic modulation while the left IC is responsible for the parasympathetic modulation (Oppenheimer et al., 1992; Napadow et al., 2008). IC is a deep brain area, and theoretically it is modulated by tDCS through common connections with the temporal cortex (TC). For example, computational modeling and experimental studies showed that tDCS applied to left TC modulated IC activity, resulting in increased parasympathetic modulation at rest and during exercise (Montenegro et al., 2011; Okano et al., 2015). Within this context, the parasympathic branch is the responsible for modulating cardiac autonomic control at rest and when exercise begins a progressive decrease in modulation is observed until its complete withdrawal.

Concerning the different brain areas stimulated, studies on tDCS show opposite results and a high variability regarding the effects on muscular strength and endurance performance. The high inter-individual variability, i.e., responders vs. non-responders, to tDCS would be a possible explanation to the variance in outcomes (López-Alonso et al., 2015). Other factors like the different electrode montages used (see Tables 1, 2) and stimulation parameters (see Tables 3, 4) also can have contributed to mixed result. Furthermore, due to differences in stimulation parameters, such as electrode size and position, even as the low focality of tDCS (Miranda et al., 2013), other brain areas beyond the target area could be affected by the electric current from tDCS, changing the results completely. Overall, tDCS seems to enhance muscular strength and endurance performances.

Although there are many differences in terms of experimental design and physical task performed, some common characteristics can be found: (i) primary motor cortex (M1) has been the most targeted area; (ii) a-tDCS was delivered main before the physical task; (iii) most of the studies applied 20 min of stimulation at 2 mA with an active electrode size of 35 cm^2^. In relation to neuromuscular parameters, a-tDCS generally increased corticospinal excitability (Cogiamanian et al., 2007; Williams et al., 2013; Hendy and Kidgell, 2014; Frazer et al., 2017). Physiological responses during exercise did not show consistent changes after a-tDCS. Notably, when perceptual responses were measured, the improvement in physical performance induced by CTEF was often associated with a lower perceived exertion (Williams et al., 2013; Okano et al., 2015; Angius et al., 2016, 2018; Lattari et al., 2018b) while muscle pain did not change. The neurophysiological mechanisms that support the effect of a-tDCS on improving physical capacity are still unclear.

With respect to resistance, Cogiamanian et al. (2007) suggested that a-tDCS could improve subjects' motivation, reduce muscular pain, and modulate muscle synergy. However, none of the proposed mechanisms and corresponding parameters were monitored. Other authors propose that the improvement in endurance performance after a-tDCS could be due to increased neural drive and a reduction in supraspinatus fatigue (Williams et al., 2013; Vitor-Costa et al., 2015). Other authors have suggested that a-tDCS could influence sensorimotor integration and associated cognitive demand without altering the motor command (Abdelmoula et al., 2016). Angius et al. (2016, 2018) proposed that, due to the increase in a-tDCS-induced corticospinal excitability, fewer excitatory stimuli for M1 were required to produce the same submaximal force or power. As perceived exertion seems to depend on excitatory inputs from the supplemental motor area (SMA) and other brain regions (de Morree et al., 2012; Zenon et al., 2015), a reduction in such inputs would result in a lower perception of effort. It should be noted, however, that two studies reported improvements in endurance performance without significant changes in corticospinal excitability (Abdelmoula et al., 2016; Angius et al., 2016). This is not surprising, since previous studies have demonstrated a considerable variability in corticospinal response after tDCS over the motor cortex (MC) (Wiethoff et al., 2014; Madhavan et al., 2016).

Studies that investigated the effects of tDCS on muscle strength indicate that performance improvement was achieved both by increased corticospinal excitability and by reduced short-interval intracortical inhibition and increased cross-activation (Hendy and Kidgell, 2014; Frazer et al., 2017). Other studies suggest that the improvement in workload was obtained by the reduction in the perception of effort (Lattari et al., 2016, 2018b). These mechanisms behind the tDCS's ergogenic effect remain unclear and should be interpreted with caution, since none of these studies monitored brain activity during exercise following tDCS.

### Limitations and Future Directions

According to the rapid increase in the tDCS studies and muscular strength and endurance performance, important methodological limitations need to be considered. The different methodological characteristics of the experiments imply caution in interpret results related to effectiveness of tDCS as ergogenic aid. The standardization of methodological variables such as montage of electrodes, current intensity, session duration and other details, is essential to provide interesting insights about the real effects of tDCS on exercise and sport performance.

In addition, the mechanisms responsible for the improvements in muscular strength and endurance performances are still unclear. In line with this, an interest question is what results in the transient improvement in muscular strength and endurance performance? It seems that the modulation of corticospinal excitability or other targeted brain areas following tDCS would be the responsible for that improvement. Nevertheless, few studies examined corticospinal or brain activity following or during tDCS. Other technicality of tDCS is the low spatial resolution of the induced electric field in the brain when compared to transcranial magnetic stimulation (TMS) (Wagner et al., 2007a,b; Miranda et al., 2013), which can affect the functioning of certain brain areas beyond the target areas. The small sample found in the studies is other important point that can increase the probability of false positive results (Button et al., 2013). Lastly, the lack of appropriate blinding methods in most studies (see Tables 3, 4) should also be considered, since unapproved blinding procedure can lead to unexpected and confounding psychological effects, making difficult the interpretation of the results (Kessler et al., 2012; Fonteneau et al., 2019).

## Conclusion

The results of this systematic review suggest that a-tDCS can improve muscular strength, but not to endurance performance. Nevertheless, evidence is insufficient to guarantee its effectiveness. New studies are required to assess the long-term effects of tDCS application combined with exercise training, whether with athletes or non-athletes. Despite tDCS is still considered a new tool in exercise and sport performance, it seems to have potential to improve performance. In line with this, more rigorous and extensive experimental studies are needed in order to better understand possible side effects from either regular use or abuse. Other important point that is needed is doing more studies with larger samples, appropriate blinding methods and techniques to examine neurophysiological mechanisms of tDCS.

## Author Contributions

SM and PJ designed the study, acquired and analyzed the data, and wrote the first draft of the paper. VA and JV helped to design the study, to organize the data acquired, and to discuss the first draft of the paper.

### Conflict of Interest Statement

The authors declare that the research was conducted in the absence of any commercial or financial relationships that could be construed as a potential conflict of interest.
